# Progress in Surgery

**Published:** 1894-10-06

**Authors:** 


### PROGRESS IN SURGERY.

### GENERAL SURGERY—*(continued)*.

**Aneurism.**—W. W. Keen[44] records a case of ligation of the common and external carotid arteries and the jugular vein for arterio-venous aneurism of the internal carotid and jugular; with division of the optic nerve on the opposite side, the result of a gunshot wound. With the exception of slight ptosis and immobility of the eyeball the case did well, but the final result as to the aneurism had not yet been ascertained. A case of traumatic aneurism of the vertebral artery successfully treated by extirpation of the sac and plugging of the seat of hæmorrhage is given by Matas.[45] Three tables of intracranial aneurisms, tramatic aneurisms, and non-aneurismal wounds and other injuries of the same vessel are commented upon. In the more common cases of wounds the difficulties of applying direct pressure to the bleeding point are less in the upper part of the neck than in the lower part, where the artery is surrounded by vital structures. Internal aneurism in European residents in Japan is the subject of an able and valuable paper by S. Eldridge.[46] It clearly brought out the fact that aneurism was much more prevalent in Japan than in any other country, and confirmed the view that syphilis was responsible for the large proportion of cases. In was possible that filarial infection had some bearing on the occurrence of aneurism. Le Dentn[47] gives an interesting case of aneurism of the brachiocephalic trunk and arch of the aorta successfully treated by ligation of the carotid and right subclavian, and four months later by ligation of the left subclavian artery. The patient could get about for eight months, when the aneurism again progressed, and terminated in death three months later. A case of ligature of both external carotids for hæmorrhage is recorded by Henry Morris,[48] who also gives a brief history of ligature of the external carotid from the time it was first performed by Bell (for a wounded vessel in removal of a tumour of the neck) up to the present. The best point on the artery for the application of the ligature is between the superior thyroid and lingual branches (Maisonneuve and Sedillot's operation). At this spot it should be ligatured—(1) For wounds of any of the branches of the vessel (except the superior thyroid) where the divided ends are not accessible; (2) for hæmorrhage from the floor of the mouth, pillars of the fauces, and base of the tongue (whether from cancer or other causes); as a preliminary step in extirpation of tumours of the parotid, antrum, soft palate and fauces, and pharynx; and for vascular tumours and cirsoid aneurism of the scalp.

Digital compression cured a case of aneurism of the common femoral artery, reported by Christopher Heath.[49] It was employed for twelve and six and a-half hours respectively, with five days interval. The treatment of aneurisms by extirpation was strongly advocated by M. Trelat at the French Surgical Congress of 1889. Recently J. Ransohoff[50] has recorded two traumatic cases in the forearm and leg, and has formulated the following conclusions regarding the operative treatment of aneurisms of the extremities: (1) Extirpation is the ideal method. It should be resorted to unless there are weighty reasons against it; (2) in aneurisms of the forearm and of the leg no other method should be adopted; (3) aneurisms which have suddenly grown large from subcutaneous rupture of the sac, and those in which rupture is impending, should be subjected to extirpation; (4) in recent traumatic aneurisms the injured vessel should be divided between two ligatures; when a sac has formed it should be excised; (5) when other methods have failed extirpation should be tried before resorting to amputation; (6) in arterio-venous aneurisms extirpation should be practised if any operation is indicated; (7) proximal ligation is to be reserved for cases of idio-pathic or spontaneous aneurisms in which the age of the patient or an enfeebled condition from other causes would make a prolonged operation hazardous, and for cases in which the position of the tumour precludes the possibility of extirpation. W. Macewen[51] decribes a mode compression of the abdominal aorta which he has employed for over fifteen years. As the patient lies on his back on the table the assistant faces his feet, standing on the left side on a line with the umbilicus. He then places his closed right hand on the patient's abdomen a little to the left of the middle line, the knuckles of the index finger just touching the upper border of the umbilicus. The assistant stands on his left foot (his right foot crossing the left and resting upon), and leans upon his right hand, thereby exercising the necessary amount of pressure. This can easily be estimated by placing the left index finger on the common femoral.

**Varicose Veins.**—For the operative treatment of varicose veins, Laplace[52] recommends ligature of the long saphenous vein at the saphenous opening, and of the short saphenous vein between the heads of the gastrocnemius. He reports seventeen cases, in six of which the operation was bilateral; cocaine anæsthesia was employed. A curious case of varicose veins on the face and tongue in a woman aged fifty-nine, and gradually increasing from the time of birth, is noted by I. R. Gridley.[53] Apart from slight spitting of blood this condition gave no trouble. Trzebicky and St. Karpinski,[54] in repeating the experiments of Braun—viz., injecting the popliteal or small saphena after ligature of the femoral vein, and finding the abdominal veins full—conclude that ligature of the femoral veins is justified when tamponage or lateral ligature would not suffice. Niebergall[55] objects to the practice of tying both the common femoral artery and vein in cases of complete or partial division of the latter vessel. Gangrene has resulted in 62·5 per cent. of the cases in which one or both vessels had been wounded during the removal of a tumour, and both vessels tied, whereas it did not occur in any one of twenty-five collected cases of ligature of the common femoral vein for wounds of this vessel caused in the removal of large growths from the thigh. Compression of the vein by antiseptic plugging, when partially divided, is not likely to be of service, except in cases of small external wound. Lateral ligature or suture of the injured vein is indicated in small longitudinal wounds in its walls. In total or almost total division complete ligature is always necessary.

[44] The Times and Register, vol. xxvii., No. 10, March 10, 1894. [45] Glasgow Med. J., Jan. 1894, p. 77, and Annals of Surgery, Nov., 1893. [46] N. Y. Med. Journal, Feb. 24, 1894, p. 185. [47] Amer. Journal Med. Sci., Feb., 1894. p. 205, and Bull. de l'Acad. de Méd. [48] The Medical Week, Jan. 5, 1894, p. 5. [49] Brit. Med. J., Feb. 10, 1894, p. 300. [50] Annals of Surgery, Jan., 1894, p. 78. [51] Annals of Surgery, Jan. 1894, p. 1, [52] Ed. Med. J., Feb., 1894, p. 762, and Phil. Med. and Surgical Reporter. [53] Med. Record, March 24, 1894, p. 366. [54] Amer. J. Med. Sci., Nov., 1893, p. 606, and archiv. für Klin. Chirurgie. [55] Ep. Brit. Med. Journal, Jan. 13, 1894, p. 6, and Deutsch. Zeitsch. f. Chirurgie.

